# Analysis of ultrasonic characteristics in 12 cases of ovarian Sertoli–Leydig cell tumour

**DOI:** 10.3389/fonc.2026.1773159

**Published:** 2026-06-23

**Authors:** Liyang Shao, Weihong Dong, Shuang Li, Qiongrui Zhao, Tingting Liu, Kaikai Li, Tian Wu, Yu Wang, Haohui Zhu, Ruili Wang

**Affiliations:** 1 Department of Ultrasound, Henan Provincial People’s Hospital, Zhengzhou, Henan, China; 2 Department of Pathology, Henan Provincial People’s Hospital, Zhengzhou, Henan, China; 3 The Department of Research and International Exchange, Henan Provincial People’s Hospital, Zhengzhou, Henan, China; 4 Department of Functional Diagnostics, The Third People’s Hospital Of Xinjiang Uygur Autonomous Region, Urumqi, Xinjiang Uygur Autonomous Region, China

**Keywords:** ovarian cancer, regional blood flow, tumour, ultrasonography, ultrasound

## Abstract

**Objective:**

To summarise the ultrasonic imaging characteristics of ovarian Sertoli–Leydig cell tumours (SLCTs) and improve the diagnostic and detection rates of this disease through ultrasound.

**Methods:**

Retrospective analysis of clinical and ultrasound data from 12 cases of SLCTs confirmed by surgery and pathology at Henan Provincial People’s Hospital between January 2015 and January 2023 was conducted. All patients underwent transabdominal or transvaginal ultrasonography. The ultrasonographic images and clinical characteristics of these 12 patients were summarised.

**Results:**

Among the 12 SLCTs, 5 (41.7%) were well-differentiated, 5 (41.7%) were moderately differentiated, 1 (8.3%) was moderately to poorly differentiated, and 1 (8.3%) was of retiform type. Seven tumours (58.3%) were solid, 3 (25.0%) were cystic-solid, and 2 (16.7%) were cystic. Internal blood flow was scored as 4 in 1 case (8.3%), 3 in 7 cases (58.3%), 2 in 2 cases (16.7%), and 1 in 2 cases (16.7%). Peripheral blood flow was scored as 4 in 8 cases (66.7%), 1 in 2 cases (16.7%), and 0 in 2 cases (16.7%). Eight cases (66.7%) showed an overall ‘surrounding ball’ blood flow pattern. Among the 9 patients with available preoperative testosterone measurements, all 9 had elevated levels.

**Conclusion:**

Ovarian SLCTs in this small retrospective series most commonly appeared as unilateral, well-circumscribed solid or cystic-solid adnexal masses, often with prominent peripheral vascularity and, in some cases, a surrounding ball Doppler pattern. These findings should be interpreted as descriptive observations rather than diagnostic criteria. In the appropriate clinical setting, particularly in young patients with hyperandrogenic manifestations, such sonographic features may raise suspicion for SLCT and prompt further diagnostic evaluation.

## Introduction

Sertoli–Leydig cell tumours (SLCTs) are a rare type of ovarian sex cord-stromal tumour, accounting for approximately 0.5% of all ovarian tumours (1). Unlike most sex cord-stromal tumours that tend to be benign, SLCTs are mostly malignant; however, their prognosis is closely related to stage, where patients with Stage I tumours have a good prognosis, while patients with advanced-stage tumours have a poorer prognosis (2). Therefore, early diagnosis is crucial. Sertoli–Leydig cell tumours predominantly occur in young women (median age approximately 30 years), and their unique clinical significance lies in frequent association with endocrine dysfunction (3–5). Since tumour cells secrete androgens, the clinical manifestations are primarily hyperandrogenaemia, including masculinising signs (hirsutism, low-pitched voice, clitoral hypertrophy) and menstrual disorders (amenorrhea). These symptoms provide important clues for clinical suspicion of SLCTs.

In imaging evaluation, ultrasound serves as the primary screening tool for ovarian tumours, valued for its real-time and convenient assessment of tumour size, morphological characteristics (solid, cystic, or mixed solid-cystic), and blood flow distribution (6–8). However, SLCTs often require differentiation from other ovarian solid tumours on ultrasound, such as theca cell tumours, which typically present with excessive oestrogen and poor blood flow, and granulosa cell tumours, characterised by the ‘Swiss cheese’ sign with moderate blood flow. Currently, due to the extremely low incidence of SLCTs, most domestic and international literature consists of case reports, lacking systematic summarisation of their ultrasound imaging features. This leads to challenges for sonographers in recognising and accurately diagnosing SLCTs when encountering atypical adnexal masses (9, 10).

Histologically, SLCTs comprise a spectrum that includes well-differentiated, moderately differentiated, poorly differentiated, and retiform variants, and some tumours may contain heterologous elements (1, 2). These histological subtypes are associated with differences in biologic behaviour and may also contribute to variation in imaging appearance.

This study conducted a retrospective analysis of clinical and ultrasound imaging data from 12 SLCT cases confirmed by surgical pathology, aiming to descriptively summarise their ultrasound imaging characteristics, including morphological and Doppler haemodynamic features, and to identify imaging patterns that may assist clinical suspicion and differential consideration of SLCTs. The findings provide ultrasound physicians with more specific diagnostic references, enhancing early detection rates and diagnostic confidence for SLCTs, thereby demonstrating significant clinical practical implications.

## Materials and methods

### Study population and diagnostic confirmation

This retrospective study included 12 patients with ovarian SLCTs diagnosed through postoperative pathology at Henan Provincial People’s Hospital (China) between January 2015 and January 2023. All cases had complete preoperative ultrasound imaging and clinical records.

The pathological diagnosis of ovarian SLCTs strictly follows the 2020 World Health Organization Classification of Tumours of the Female Genital System (11). Two senior pathologists at our hospital independently reviewed the histological sections and jointly confirmed the diagnosis. Tumours are classified into four grades based on differentiation: well-differentiated, moderately differentiated, poorly differentiated, and reticulum-type.

### Clinical data collection

The following data were collected for all patients by reviewing the hospital’s electronic medical record system: ① demographic data (age); ② clinical symptoms and signs; ③ preoperative serum sex hormone levels (focusing on testosterone); ④ surgical procedures; ⑤ postoperative pathological reports (including tumour size, laterality, pathological type). This study protocol was reviewed and approved by the Ethics Committee of Henan Provincial People’s Hospital (Approval No.: 2021LSNo.214), and the requirement for informed consent from patients was waived.

### Ultrasound equipment

The instruments used in this study included the SIEMENS S2000, GE Voluson S8, and GE Voluson E10 colour Doppler ultrasound diagnostic systems. Transducers included the following: a 2D convex array probe 4C1, with a frequency ranging between 1–4.5 MHz; C1-5, with a frequency ranging between 2–5 MHz; an endocavitary probe (RIC6-12-D) with a frequency ranging between 5–13 MHz.

All ultrasound examinations were performed by physicians with at least 5 years of experience in gynaecological ultrasound diagnosis.

### Transabdominal and transvaginal ultrasound examination protocols

The examination process followed standardised scanning protocols as follows.

In the transabdominal ultrasound examination (TAUS), the patient was positioned in the supine position with the bladder moderately full. A convex array probe was used for scanning. First, in the sagittal (longitudinal) view of the uterus, the uterine position, size, homogeneity of myometrial echogenicity, endometrial thickness, and morphology were comprehensively assessed. Then, the probe was rotated 90° to perform horizontal (transverse) scanning of the uterus, sliding continuously from the uterine fundus to the cervical level to observe the transverse diameter and bilateral uterine horns. Finally, the adnexal regions were focused on. The probe was placed obliquely in the iliac fossa region, and bilateral ovaries were examined from multiple angles using oblique and horizontal planes. Once an adnexal mass was identified, the following values were systematically recorded. The maximum 3 dimensions of the mass were measured, and its shape (e.g. round, oval, lobulated) was described to record the size and morphology. The clarity of the mass border, wall thickness, and internal echo characteristics (solid hypoechoic, cystic anechoic, mixed echo) and their homogeneity were assessed to describe the border and internal echogenicity of the tumour. Colour Doppler flow imaging (CDFI) was used to observe and record the richness of blood flow signals within and around the mass and the distribution pattern (e.g. peripheral ring-like, internal cord-like). The anatomical relationship of the mass with the ipsilateral ovary, uterus, and pelvic wall was also assessed.

For the transvaginal ultrasound examination, the patient was positioned in the lithotomy position with the bladder emptied. A transvaginal probe (5–13 MHz) was used for scanning. First, the uterine sagittal view was obtained to observe the myometrium, endometrium, and cervix in detail. Then, the uterine transverse view was obtained to assess the uterine cavity shape and bilateral cornua. A systematic scan of the bilateral adnexal regions was performed. By multi-angular rotation and tilting of the probe, the ovarian structures were carefully identified in the ovarian long-axis and short-axis views. Upon identifying a lesion, a detailed assessment was performed to document lesion characteristics as per TAUS, recording the size, morphology, border, and internal echogenicity in detail. Pulsed wave Doppler and CDFI were used to assess blood flow distribution, grading (according to the aforementioned criteria), and the resistance index (RI) within and in the solid components at the periphery of the lesions were measured. Then, the probe was gently pushed to assess the relative movement (mobility) of the mass with surrounding tissues (e.g. ovary, uterus) to make a preliminary adhesion judgement. All image storage and measurements were performed using the device’s built-in workstation.

### Classification criteria of mass properties

Based on the definition of mass composition in the American College of Radiology’s Ovarian-Adnexal Imaging Reporting and Data System (O-RADS), ultrasound risk stratification and management consensus (3), as well as the incorporation of two-dimensional ultrasound imaging features, the masses were classified into 3 categories as follows.

Solid mass: where solid components in the mass accounted for more than 80%, and cystic components could be ignored or were absent.

Cystic mass: where the mass was purely cystic, or the solid component could be ignored (such as only a thin wall, fine septum, irregular inner wall, and a small amount of papilla).

Solid cystic mass: where the mass contained both identifiable solid and cystic components, and the solid component represented less than 80%.

The nature of the mass in all cases was independently judged by two ultrasound physicians who were unaware of the pathological results, and a consensus was reached when there was no agreement.

### Blood flow RI measurement standard

Pulsed wave Doppler was used to measure the RI. The sample volume was set to 2 mm, with a correction angle <60°. At least 3 consecutive, stable Doppler waveforms were recorded, and the average value was taken as the final RI.

Measurements followed the standards below.

Peripheral RI: measured in the solid area at the mass periphery or at the edge of the solid part of a cystic-solid mass, selecting the largest and brightest feeding vessel.

Internal RI: measured within the solid component of the mass, selecting areas with continuous blood flow signals, and avoiding areas with obvious calcification, necrosis, or no flow. If multiple measurable blood flows were present within or around the mass, the lowest RI value was recorded.

### Statistical methods

Statistical analysis was performed using SPSS 23.0 (IBM Corp., Armonk, NY, USA). As this study was retrospective descriptive research using a small sample size (n=12), all statistical analyses were primarily descriptive. Data normality testing: The Shapiro–Wilk test was first applied to assess the normality of all quantitative variables (e.g. age, tumour maximum diameter, RI). Quantitative data: Variables conforming to normal distribution were expressed as mean ± standard deviation (x ± s); those not conforming to normal distribution or with small sample sizes were presented as median (interquartile range) (*M* [*P*25, *P*75]). In this study, patient age, tumour size, and blood flow RI are described using median (interquartile range). Discrete data: Frequency (percentage) (n [%]) was used for representation. Furthermore, pathological type, mass nature (solid/cystic-solid/cystic), blood flow score (0–4), incidence of ‘surrounding ball’ sign, clinical manifestations, and other values are described using this method.

## Results

### General characteristics

A total of 12 patients were included in this study. The age range was 15–46 years, with a median of 32 (28–36) years and a mean of (32.5 ± 8.9) years. All masses were unilateral: 6 were left-sided (50.0%), and 6 were right-sided (50.0%). The maximum lesion diameter ranged from 1.7 to 23.0 cm, with a median of 4.5 (2.6–6.1) cm and a mean of (6.8 ± 6.1) cm. All cases were confirmed by postoperative pathology.

The pathological types of the 12 ovarian SLCTs were: 5 well-differentiated (41.7%), 5 moderately differentiated (41.7%), 1 moderately to poorly differentiated (8.3%), and 1 retiform type (8.3%). The results concerning classification by O-RADS criteria for mass nature were as follows: 7 solid (58.3%), 3 cystic-solid (25.0%), and 2 cystic (16.7%). Ascites was present in 1 case (8.3%).

In terms of clinical manifestations, among the 12 patients, 7 cases (58.3%) exhibited masculinising features (such as hirsutism, low-pitched voice, and clitoral hypertrophy), 6 cases (50.0%) showed feminising features (including amenorrhea and oligomenorrhea), and 3 cases (25.0%) displayed feminising characteristics (such as increased menstrual flow and irregular bleeding). Some patients exhibited multiple manifestations simultaneously. Among the 9 patients with available preoperative serum testosterone levels, all 9 had values exceeding the normal reference range (<0.74 ng/mL).

Regarding blood flow scoring, internal blood flow was observed as follows: 4 points (rich) in 1 case (8.3%), 3 points (striped) in 7 cases (58.3%), 2 points (spotted) in 2 cases (16.7%), and 1 point (no blood flow) in 2 cases (16.7%). For peripheral blood flow, there were: 4 points (rich) in 8 cases (66.7%), 1 point (no blood flow) in 2 cases (16.7%), and 0 points (not detected) in 2 cases (16.7%). A total of 8 cases (66.7%) exhibited the ‘ball-holding sign’ overall. The median RI for internal blood flow was 0.47 (0.33–0.65), while the median RI for peripheral blood flow was 0.55 (0.39–0.60).

The detailed clinical characteristics, treatment methods, prognoses, and ultrasonic features of 12 patients are shown in **Tables 1**, **2**.

**Table 1:** Clinical characteristics, management, and prognosis of 12 ovarian SLCTs patients in this cohort.

Number	Age (years)	Clinical symptom	Testosterone (0.74ng/ml)	Pathological type	Modus operandi	Follow-up results
1	38	One-year amenorrhea, clitoral hypertrophy, coarse voice, facial and back acne, and small beard around the mouth	4.3	Semi-differentiated	Bilateral ovariectomy and hysterectomy	Follow-up for 7 years and 8 months without recurrence
2	32	Male facial features, inverted nipples, small beard around mouth, clitoral enlargement/infertility	4.7	High differentiation	Tumour Excision	Follow-up for 3 years and 6 months without recurrence
3	15	She has had infrequent menstruation for 1 year with a 2-month cycle and amenorrhea for 5 months, accompanied by facial acne and hirsutism.	1.6	reticular pattern	Tumour Excision	Patient death
4	46	Irregular menstruation for 3 years, irregular vaginal bleeding	2.1	High differentiation	Tumour Excision	Follow-up for 6 years and 9 months without recurrence
5	28	She has been amenorrhoeic for two years, with clitoral hypertrophy and hirsutism, acne on the face and back, and a small beard around the mouth.	4.2	High differentiation	Tumour Excision	No recurrence after 5 years and 1 month of follow-up
6	28	A menstrual cycle lasting 2–3 months, accompanied by increased hair growth, a coarse voice, and clitoral enlargement.	3.9	Semi-differentiated	Tumour Excision	Follow-up for 4 years and 7 months without recurrence
7	24	Two years of amenorrhea, acne on the face and back, hoarse voice, and a small beard around the mouth	4.5	Semi-differentiated	Tumour Excision	Follow-up for 3 years and 2 months with no recurrence
8	33	Menstrual cycle 2–3 months, less flow.	5.2	Semi-differentiated	Tumour Excision	Follow-up for 2 years and 10 months showed no recurrence
9	36	Infrequent menstruation with a 2-month cycle, hirsutism, small facial hair around the mouth, and acne on the face and back.	3.7	High differentiation	Tumour Excision	The follow-up was 1 year and 9 months, and there was no recurrence
10	21	The menstrual cycle is shortened by 2 years	not have	High differentiation with partial cystic changes	Tumour excision	Follow-up for 1 year and 6 months with no recurrence
11	42	Menstruation delayed by 2 months with heavy menstrual flow	not have	Medium-low differentiation	Three months after the tumour excision, the uterus and both adnexa were removed	No recurrence after 1 year and 7 months of follow-up
12	45	Heavy menstrual bleeding with dysmenorrhea for 1 year	not have	moderately differentiated	Three months after the tumour excision, the uterus and both adnexa were removed	No recurrence after 8 months of follow-up

**Table 2:** Ultrasound features of 12 ovarian SLCTs patients in this group.

Number	Test mode	Affected ovary	Diameter (cm)	Ultrasonic properties	Ultrasonic blood flow characteristics	Perioperative blood flow score	Peripheral blood flow resistance	Internal blood flow score	Internal blood flow resistance
1	Ultrasound	left side	2.6	Solid	Peripheral blood flow shows ball-hugging sign, and internal blood flow is relatively rich	4	0.39	3	0.33
2	Ultrasound	right side	2.5	Solid	Peripheral blood flow shows ball-hugging sign, and internal blood flow is relatively rich	4	0.6	3	0.38
3	belly	right side	23	Cystic Multisegmental	There is a small amount of blood flow around the wall, and the septum has a rich blood flow	1	0.5	3	0.43
4	Ultrasound	right side	1.5	Solid	No blood flow around, with minimal blood flow inside	0		2	0.56
5	belly	left side	5.9	Solid	Peripheral blood flow shows ball-hugging sign, and internal blood flow is relatively rich	4	0.52	3	0.42
6	belly	left side	6.1	Solid	Peripheral blood flow shows a ball-hugging sign, and internal blood flow is abundant	4	0.6	4	0.54
7	belly	right side	6.7	Cystic Multisegmental	Peripheral blood flow shows a ball-hugging sign with minimal blood flow across the diaphragm	4	0.6	2	0.65
8	Ultrasound	left side	2.7	Solid	Peripheral blood flow shows ball-hugging sign, and internal blood flow is relatively rich	4	0.59	3	0.45
9	Ultrasound	right side	3	Solid	Peripheral blood flow shows ball-hugging sign, and internal blood flow is relatively rich	4	0.5	3	0.45
10	Transabdominal	left side	3.1	Solid	The periphery has abundant blood flow and presents the ball-hugging sign, while the interior has relatively abundant blood flow	4	0.52	3	0.47
11	Ultrasound	left side	3.9	Solid	No blood flow signal in the surrounding area or inside	1		1	
12	Ultrasound	left side	1.7	Solid	Abundant peripheral blood flow with a ball-holding sign, minimal internal blood flow	4	0.57	2	0.43

### Ultrasound manifestations of ovarian Sertoli–Leydig cell tumours

Based on ultrasound results, the following were observed for SLCTs.

**Figure 1:**
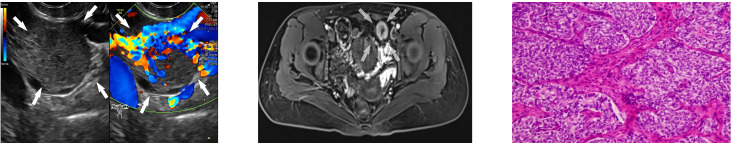
A 38-year-old patient with back acne and menstrual irregularities was diagnosed with a solid left ovarian Sertoli-Leydig cell tumor. Ultrasound findings (indicated by thick arrows): hypoechoic with regular morphology, well-defined margins, and heterogeneous internal echoes. Peripheral ring-shaped blood flow signals and internal linear flow signals were observed. Contrast-enhanced MRI (indicated by thin arrows) showed non-uniform ring enhancement. Pathological examination confirmed a moderately differentiated Sertoli-Leydig cell tumor.

Solid (7 cases, 58.3%): The median tumour diameter was 2.7 cm (2.5–3.1 cm), with 3 cases on the left side and 4 on the right. The masses typically exhibited homogeneous or heterogeneous hypoechoic patterns with regular shapes and well-defined borders. Colour Doppler flow imaging revealed coarse ring-shaped blood flow signals (4 points) around the lesions in 6 cases (85.7%), accompanied by slightly abundant linear blood flow signals (3 points) within (**Figure 1**). Among these, 6 cases (85.7%) exhibited a typical surrounding ball blood flow distribution. Pulsed wave Doppler detection showed that peripheral arterial flow spectra were mostly low resistance.

**Figure 2:**
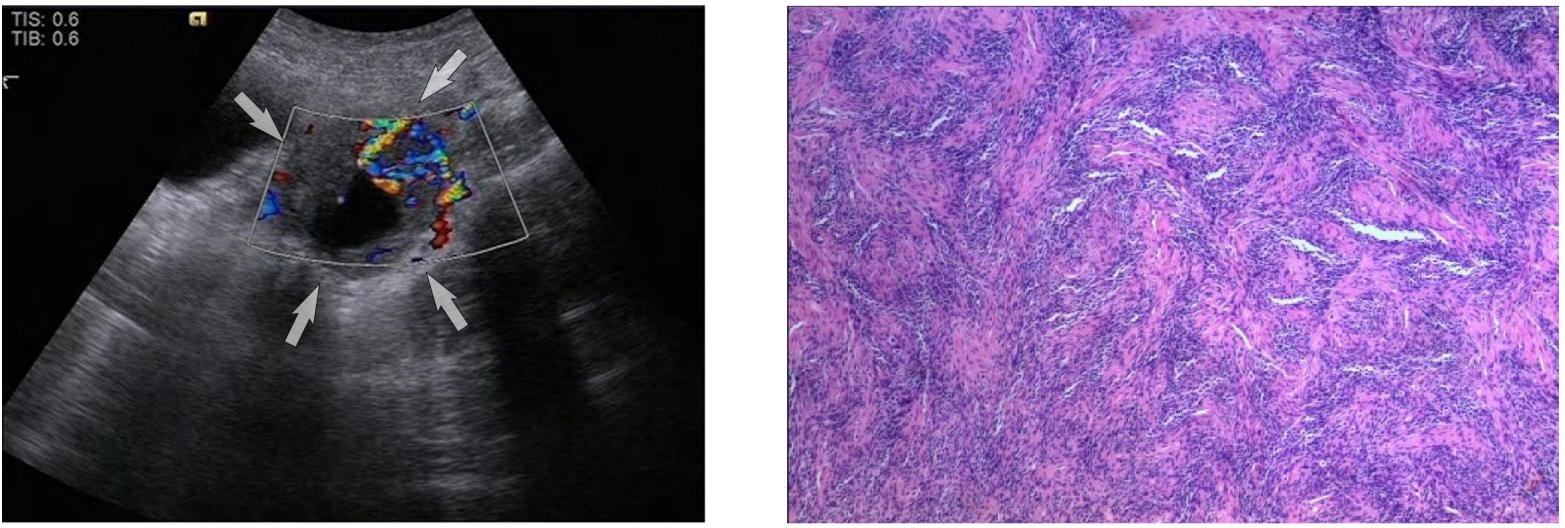
A 28-year-old patient presenting with “menstrual disorders and infertility” was diagnosed with a cystic-solid Sertoli-Leydig cell tumor in the left ovary (arrows). The mass exhibited mixed echogenicity, predominantly solid hypoechoic with regular morphology and well-defined borders. The internal echogenicity was heterogeneous, showing scattered small cystic anechoic areas. Transillumination revealed acceptable internal acoustic transmission. CDFI demonstrated coarse peripheral ring-shaped blood flow signals, with slightly abundant linear blood flow signals within the solid hypoechoic area. Pathological examination confirmed a moderately differentiated Sertoli-Leydig cell tumor.

Cystic-solid (3 cases, 25.0%): The median tumour diameter was 6.0 cm (5.9–6.1 cm), with 3 cases located on the left side. The masses exhibited mixed echogenicity, predominantly solid hypoechoic with scattered small cystic anechoic areas, and well-defined borders. Colour Doppler flow imaging: 2 cases showed thick ring-like peripheral flow (Score 4), with moderately abundant strip-like flow (Score 3) in the solid parts; 1 case showed no significant flow (Score 1) either peripherally or internally. Pulsed wave: In lesions where flow was detected, peripheral arterial spectra were low resistance, while internal arterial spectra showed medium resistance (**Figure 2**). The surrounding ball sign was present in 2 cases.

**Figure 3:**
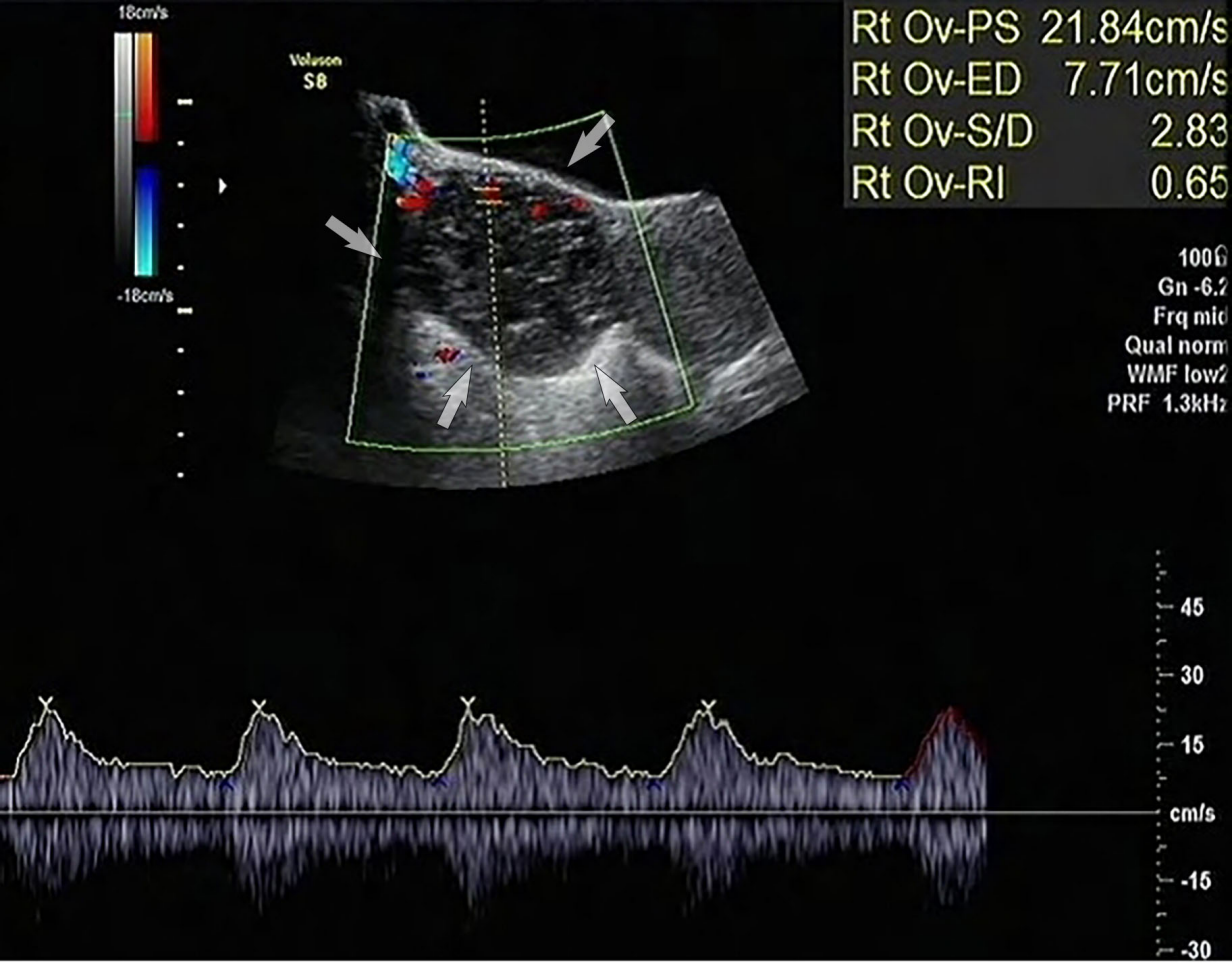
A 23-year-old female patient presented with vaginal bleeding and was diagnosed with a cystic right ovarian Sertoli-Leydig cell tumor (arrows). The mass was hypoechoic with thin walls and smooth inner surfaces, containing numerous fine septa. CDFI showed no significant blood flow signals in the cyst wall, but moderate resistance blood flow signals were observed above the septa. Pathology confirmed a moderately differentiated Sertoli-Leydig cell tumor.

Cystic (2 cases, 16.7%): The median tumour diameter was 14.85 (6.7–23.0 cm), all located on the right side. Masses were anechoic, with thin and smooth inner walls, and contained numerous fine septations. Colour Doppler flow imaging: No significant flow was seen in the cyst wall; punctate or strip-like flow signals (Score 2–3) were seen on the septa. Pulsed wave: Flow spectra on the septa showed medium resistance (**Figure 3**). One case exhibited a surrounding ball blood flow distribution.

## Discussion

Sertoli–Leydig cell tumours are rare ovarian sex cord-stromal tumours that often occur in younger patients and may present with endocrine manifestations related to androgen excess (4, 7, 11–13). In this retrospective case series of 12 pathologically confirmed tumours, we descriptively summarised sonographic morphology and Doppler findings related to these tumours. Most lesions appeared as unilateral solid or cystic-solid masses with relatively well-defined margins, and a substantial proportion showed prominent peripheral vascularity, including a surrounding ball Doppler pattern. These observations may be useful for pattern recognition but should not be interpreted as diagnostic criteria.

Demidov et al. (10) reported that SLCTs were predominantly solid (53.3%) or cystic-solid (40.0%) on ultrasound, which is broadly consistent with our findings. In our series, solid tumours accounted for a slightly higher proportion, whereas purely cystic lesions were less frequent. This discrepancy may be related to differences in sample size and the distribution of pathological types in the enrolled cases. Notably, the aforementioned study did not provide a detailed scoring of blood flow or describe the characteristic surrounding ball sign. In our series, the surrounding ball Doppler pattern was observed in a substantial proportion of cases. However, given the lack of a control group and comparative validation, this finding should be interpreted as a descriptive observation that may raise suspicion for SLCT rather than as a specific diagnostic sign. Some studies have noted that testicular Leydig cell tumours also exhibit a similar surrounding ball appearance, characterised by peripheral ring-like blood flow perfusing towards the centre (12–15). Considering that the Leydig cells in ovarian SLCTs are homologous to testicular Leydig cells, they may share similar blood perfusion patterns, which could be the pathological basis for the surrounding ball sign. In this study, tumour blood flow resistance was mostly low to medium, consistent with the hypervascular nature of these tumours.

In this study, 3 patients underwent magnetic resonance imaging (MRI) examinations simultaneously, and the findings were consistent with previous research (16, 17). The solid components showed high signal intensity on diffusion-weighted imaging and significant enhancement after contrast administration. Ultrasound should not, however, be considered superior to MRI based on the present study’s results. Rather, ultrasound serves as an accessible first-line modality with unique advantages in the real-time assessment of morphology and Doppler haemodynamics, whereas MRI provides complementary tissue characterisation. The diagnostic contribution of ultrasound is also operator-dependent. One case in this study demonstrated no detectable blood flow signals either peripherally or intrinsically in a cystic-solid mass, and a histopathological classification as moderately to low differentiated. This suggests that even poorly differentiated SLCTs may exhibit atypical blood flow patterns, requiring comprehensive clinical and imaging assessments to avoid diagnostic exclusion due to insufficient blood flow (4, 18, 19).

Regarding differential diagnosis, SLCTs must be distinguished from other ovarian sex cord-stromal tumours. Thecomas are mostly solid but often have sparse internal blood flow signals (20, 21). Granulosa cell tumours can present as solid or cystic-solid, but their characteristic ‘Swiss cheese’ sign (multiple small internal cystic spaces) differs from the ultrasonographic appearance of SLCTs, and their blood flow richness is usually lower than that of SLCTs (21) (see the differential table in the text). Therefore, when ultrasound reveals a hypervascular solid or cystic-solid adnexal mass, especially when the blood flow distribution shows a surrounding ball sign, and the patient presents with clinical symptoms of hyperandrogenism, the possibility of SLCTs should be highly suspected. To more clearly present the characteristics of SLCTs summarised in this study, and the typical features of other tumours from the literature requiring differentiation, these key points are summarised below (see **Table 3**). This table integrates the findings of this study with existing literature reports to provide a practical reference framework for clinical ultrasonic differential diagnosis.

**Table 3:** Comparison of ultrasound features and key differentiation criteria for common ovarian tumours based on 12 SLCT cases in this study.

Tumour type	Shape regularity	Border clarity	Tumour nature	Internal blood flow classification	Internal blood flow resistance index (RI) range	Perioperative blood flow classification	Peripheral blood flow resistance index (RI) range	Specific signs
Ovarian Sertoli-Leydig cell tumour (10)	All rules	Multiple borders are clearly defined	Can be cystic, solid, or mixed	Most are strip blood flow signals (Level 3)	0.33~0.65(0.47 ± 0.11)	Rich blood flow, mostly grade 4	0.39~0.60(0.55 ± 0.07)	The blood flow around the ball is abundant, showing the “ball-hugging sign”
theca cell tumour (22,23)	Most rules	Multiple borders are clearly defined	Most are solid, a small number are cystic-solid, and no cystic	Most of the blood flow signals are strip or star-shaped (level 1-2)	0.32~0.65(0.49 ± 0.09)	Low blood flow, usually grade 1-2	0.32~0.79(0.55 ± 0.15)	Internal sound attenuation is available
granular cell tumour (21– 22)	Most rules	Multiple borders are clearly defined	The smaller ones are mostly solid; the larger ones are mostly cystic; some are cystic-solid	Mostly moderate flow signals (grade 2-3)	0.37~0.86(0.52 ± 0.12)	Low blood flow, usually grade 1-2	0.3~0.75(0.56 ± 0.16)	The presence of cystic components may present as a “Swiss cheese sign”
Other sex cord stromal tumours(22)	Most rules	Multiple borders are clearly defined	Most are solid, a small number are cystic	Most of them are medium blood flow signals (level 2-3)	0.39~0.68(0.54 ± 0.14)	Low blood flow, mostly grade 1	Cannot measure	Few specific signs

This table provides a reference framework for clinical ultrasound differential diagnosis, but it is not derived from large sample control studies or authoritative guidelines. In practical application, it should be combined with the specific situation of patients for comprehensive judgment.

From a practical perspective, these findings of the current study may be considered within the O-RADS framework as features of a vascular adnexal mass with a solid or solid-cystic component. Although our data do not justify proposing a specific risk category for SLCTs, recognition of this combination of morphology and Doppler vascularity (particularly in patients with hyperandrogenic symptoms or elevated testosterone) may prompt more focused endocrine correlation and appropriate surgical-pathological evaluation.

This study has some limitations. First, although the sample size was expanded to 12 cases, it remains relatively small for considering a rare tumour, which may affect the generalisability and representativeness of some statistical characteristics. Second, this was a retrospective single-centre study, and the imaging findings were not assessed in a blinded comparative design. Third, no control group of other hypervascular ovarian tumours was included; therefore, the specificity, sensitivity, reproducibility, and diagnostic performance of the observed Doppler patterns could not be evaluated. Fourth, not all clinical laboratory variables were available for all of the included patients. Finally, molecular and genetic data, including DICER1 status, were not systematically available, precluding the analysis of possible associations between genotype and imaging phenotype. Future research should involve larger, multicentre prospective studies combined with molecular pathological indicators to further validate and refine the imaging characteristics of SLCTs.

In conclusion, in this small retrospective case series, ovarian SLCTs most often appeared as unilateral solid or cystic-solid adnexal masses with relatively rich peripheral vascularity, and some lesions showed a surrounding ball Doppler pattern. These sonographic findings, when considered together with young age and hyperandrogenic manifestations, may help raise suspicion for SLCT. However, the present results are exploratory and descriptive in nature only, and pathological confirmation remains essential.

## Data Availability

The original contributions presented in the study are included in the article/supplementary material. Further inquiries can be directed to the corresponding authors.
